# IL-6 and TGF-β-Secreting Adoptively-Transferred Murine Mesenchymal Stromal Cells Accelerate Healing of Psoriasis-like Skin Inflammation and Upregulate IL-17A and TGF-β

**DOI:** 10.3390/ijms241210132

**Published:** 2023-06-14

**Authors:** Nerea Cuesta-Gomez, Laura Medina-Ruiz, Gerard J. Graham, John D. M. Campbell

**Affiliations:** 1 Chemokine Research Group, Institute of Infection, Immunity and Inflammation, University of Glasgow, 120 University Place, Glasgow G12 8TA, UK; cuestago@ualberta.ca (N.C.-G.);; 2 Tissues, Cells and Advanced Therapeutics, The Jack Copland Centre, Scottish National Blood Transfusion Service, Currie EH14 4AP, UK

**Keywords:** mesenchymal stromal cell, psoriasis, MSC secretome, cytokines, chemokines, IL-6, TGF-β, IL-17A

## Abstract

Mesenchymal stromal cells (MSC) show promise as cellular therapeutics. Psoriasis is a chronic inflammatory disease affecting the skin and the joints. Injury, trauma, infection and medications can trigger psoriasis by disrupting epidermal keratinocyte proliferation and differentiation, which activates the innate immune system. Pro-inflammatory cytokine secretion drives a T helper 17 response and an imbalance of regulatory T cells. We hypothesized that MSC adoptive cellular therapy could immunomodulate and suppress the effector T cell hyperactivation that underlies the disease. We used the imiquimod-induced psoriasis-like skin inflammation model to study the therapeutic potential of bone marrow and adipose tissue-derived MSC in vivo. We compared the secretome and the in vivo therapeutic potential of MSC with and without cytokine pre-challenge (“licensing”). The infusion of both unlicensed and licensed MSC accelerated the healing of psoriatic lesions, and reduced epidermal thickness and CD3^+^ T cell infiltration while promoting the upregulation of IL-17A and TGF-β. Concomitantly, the expression of keratinocyte differentiation markers in the skin was decreased. However, unlicensed MSC promoted the resolution of skin inflammation more efficiently. We show that MSC adoptive therapy upregulates the transcription and secretion of pro-regenerative and immunomodulatory molecules in the psoriatic lesion. Accelerated healing is associated with the secretion of TGF-β and IL-6 in the skin and MSC drives the production of IL-17A and restrains T-cell-mediated pathology.

## 1. Introduction

Psoriasis is a chronic inflammatory disease with a prevalence rate of 2–3% of the world population and up to 11% in some Northern European countries. Psoriasis involves the skin but can also involve the joints, leading to psoriatic arthritis, and the sclera, resulting in scleritis [1,2,3,4]. Histologically, psoriasis is characterized by epidermal hyperplasia, leukocyte infiltration, and increased dermal vascularity [5]. The initiation of psoriatic disease is poorly understood but can be triggered by several factors including injury, trauma, infection and medications that result in the dysregulation of epidermal keratinocytes. Dysregulated keratinocytes in lesional psoriatic skin release extracellular lipids and CCL20 [6], which activates dendritic cells, leading to self-antigen presentation as well as the secretion of pro-inflammatory cytokines that promote T cell activation, recruitment and expansion [7]. Activated T cells migrate to the epidermis, further promoting epidermal hyperproliferation and activation of keratinocytes, thereby contributing to the amplification of the local immune response [8,9,10]. IFN-γ-and IL-17A have been described as potential drivers of disease, causing flares in resolved psoriatic lesions [11,12]. Moreover, Bovenschen et al. described a dysfunction of regulatory T cells in psoriasis as they differentiate towards IL-17-expressing Tregs, promoting exacerbated chronic inflammation [13].

The potential of mesenchymal stromal cells (MSC) to immunomodulate and suppress effector T cell activities provides a rationale for the clinical use of MSC in diseases in which T cell hyperactivation plays a role. There are a variety of case reports [14,15,16,17,18,19,20,21,22,23,24] which show potential for MSC in the treatment of psoriasis, but the number of patients treated is very small, and all studies use different MSC preparations-in cell tissue source, cultivation and autologous versus allogeneic donation. This variability is common in the MSC therapy field and makes comparisons between studies extremely difficult. Bone marrow (BM) MSC are considered the gold standard and are the most used MSC in the clinic; however, their isolation requires an invasive and painful process [25]. Adipose tissue (Ad) is considered clinical waste from liposuctions, its obtention implies no burden to the donor and holds 500-fold more MSC than bone marrow, making adipose tissue a suitable source for the isolation of MSC for clinical use [26,27,28,29,30]. Herein, we aim to compare the immunoregulatory potential of BM and Ad MSC for the treatment of psoriasis using the well-characterized imiquimod (IMQ)-induced psoriasis-like skin inflammation model. Several authors have previously shown the efficacy of human MSC-based cell therapies in IMQ-induced murine models of psoriasis [15]; however, the xenoimmunity associated with this approach is a major barrier to elucidate the immunoregulatory mechanisms of MSC for the alleviation of psoriasis.

Pre-challenging MSC before therapeutic administration using inflammatory cytokines-known as “licensing” can be used as a method to amplify their anti-inflammatory function by increasing the expression of key immunoregulatory molecules, e.g., hepatocyte growth factor (HGF) and TGF-β [31,32]. IFN-γ treatment of MSC in combination with TNF-α, and IL-1β produces a synergistic effect, decreasing expression of pro-inflammatory factors while increasing the anti-inflammatory function of MSC [31,32,33]. The exact benefit of this pre-programming remains controversial, and a variety of in vivo studies support or reject this as a strategy on a spectrum from “essential for function” right through to having a detrimental effect [31,34,35,36,37,38,39]. The study of these complex immune interactions can be further confounded by the use of human MSC, e.g., in murine models where xeno-recognition and potential rejection adds a layer of complexity not found in the use of allogeneic donor cells in humans [40,41]. 

Herein, we have used highly characterized autologous and sex-matched mouse bone marrow-(BM) and adipose-derived (Ad) MSC for our IMQ-induced psoriasis-like skin inflammation model. Furthermore, we compared the infusion of an unlicensed control and licensed MSC to understand the role of licensing in the treatment of IMQ-induced psoriasis-like skin inflammation. In addition, we have correlated the differential pro-resolution potential observed in our in vivo model with the differential transcription and secretion profile of MSC under unlicensed and licensed conditions. 

## 2. Results

### 2.1. MSC Infusion Alleviates Imiquimod-Induced Psoriasis-like Skin Inflammation

**Figure 1:**
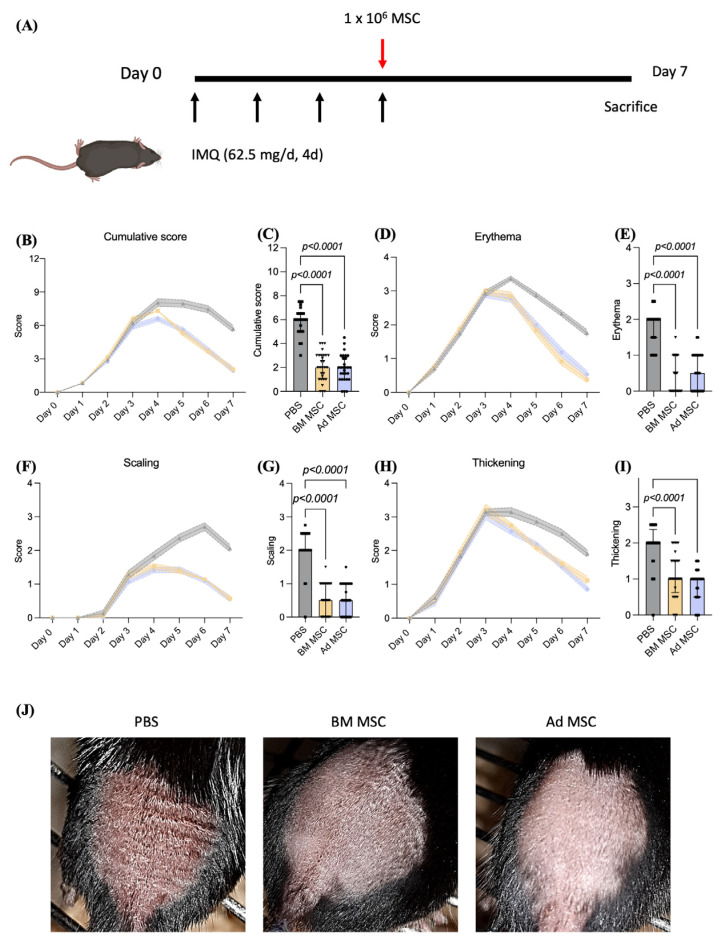
MSC infusion ameliorated psoriatic symptoms in IMQ-treated mice. (**A**) Experimental protocol showing treatment regimen using MSC in psoriatic mice. Briefly, 7-week-old C57BL/6 female mice with an average weight of 19.0 ± 1.0 g received 62.5 mg Aldara cream containing 5% IMQ on their shaved backs for four consecutive days. Black arrows represent IMQ application. The effects of MSC were tested by administration of 1 × 10^6^ BM (yellow) or Ad MSC (blue) in 0.1 mL of sterile PBS or sterile PBS alone (control mice, grey) via the tail vein on the last day of IMQ treatment (represented with a red arrow); 96 h later, mice were euthanized using CO_2_ followed by femoral artery exsanguination and skin samples were collected. Psoriasis Area and Severity Index (PASI) was applied daily to objectively score the severity of inflammation of the back skin; (**B**) the cumulative score served as a measure of the psoriasis-like severity (0 to 12). (**D**) erythema, (**F**) scaling and (**H**) thickness were scored on a scale from 0 to 4 as follows: 0, none; 1, slight; 2, moderate; 3, severe and 4, very severe. PASI scored on day 7 graphed for (**C**) cumulative score, (**E**) erythema, (**G**) scaling and (**I**) thickening. (**J**) Typical presentation of the mouse back skin 7 days after first IMQ application. Five mice were used per experimental group and experiment was repeated three times. Data shown is the cumulative of the three independent experiments. Kruskal–Wallis test (also known as one-way ANOVA) coupled with Dunn’s multiple comparisons was performed to analyze statistical significance.

IMQ was topically applied to the back of the mice daily for four consecutive days prior to intravenous (IV) administration of BM or Ad MSC (1 × 10^6^ cells/mouse in 100 μL of PBS) or PBS and mice were monitored daily for skin symptoms (Figure 1A); 24 h after initial IMQ application, the skin on the back of the mice displayed typical symptoms of erythema (Figure 1B), scaling (Figure 1D) and thickening (Figure 1F), which increased in severity over 4 days. On day 3, prior to the last IMQ application and IV injection of MSC, the combined PASI scores were between 5.88 and 6.57 for all the groups, with no significant differences among them. On day 4, 24 h after the last IMQ application (+/− MSC infusion), the PASI score stopped increasing and the skin started to heal in all groups, as represented by the gradual decrease of the cumulative PASI score (Figure 1H). PBS-injected mice healed slowly and had a median cumulative PASI score of 6.0 (IQR 5.0–6.5) on day 7 when the experiments were terminated, compared to 2 (IQR 1.0–3.0; *p* < 0.0001) for BM MSC treated mice and 2 (IQR 1.0–2.8; *p* < 0.0001) for Ad MSC treated mice (Figure 1C). Similarly, erythema decreased from 2 (IQR 1.5–2.0) in PBS-infused mice to 0 (IQR 0.0–1.0; *p* < 0.0001) and 0.5 (IQR 0.0–1.0; *p* < 0.0001) in BM and Ad MSC-treated mice, respectively (Figure 1E). In addition, scaling decreased from 2.0 (IQR 2.0–2.5) in PBS-infused mice to 0.5 (IQR 0.0–1.0; *p* < 0.0001) in both BM and Ad MSC-treated mice (Figure 1G), while thickening decreased from 2.0 (IQR 2.0–2.3) in PBS-infused mice to 1.0 (IQR 0.6–1.5; *p* < 0.0001) and 1.0 (IQR 0.5–1.0; *p* < 0.0001) in BM and Ad MSC-treated mice, respectively (Figure 1I). The typical presentation of the skin of mice infused with control PBS, BM or Ad MSC 7 days after first IMQ application (Figure 1J). PBS-infused mice showed signs of redness and flakiness as well as ridged or undulating skin due to increased thickness in the affected skin. BM or Ad MSC-infused mice, on the contrary, showed no signs of redness or scaling and the skin surface was smooth.

In summary, the infusion of BM or Ad MSC resulted in the accelerated alleviation of IMQ-induced psoriasis-like inflammation measured as a reduced PASI score.

### 2.2. MSC Infusion Reduces Epidermal Thickness and CD3^+^ Cell Infiltration through the Upregulation of Cytokines and the Downregulation of Keratinocyte Differentiation Markers

**Figure 2:**
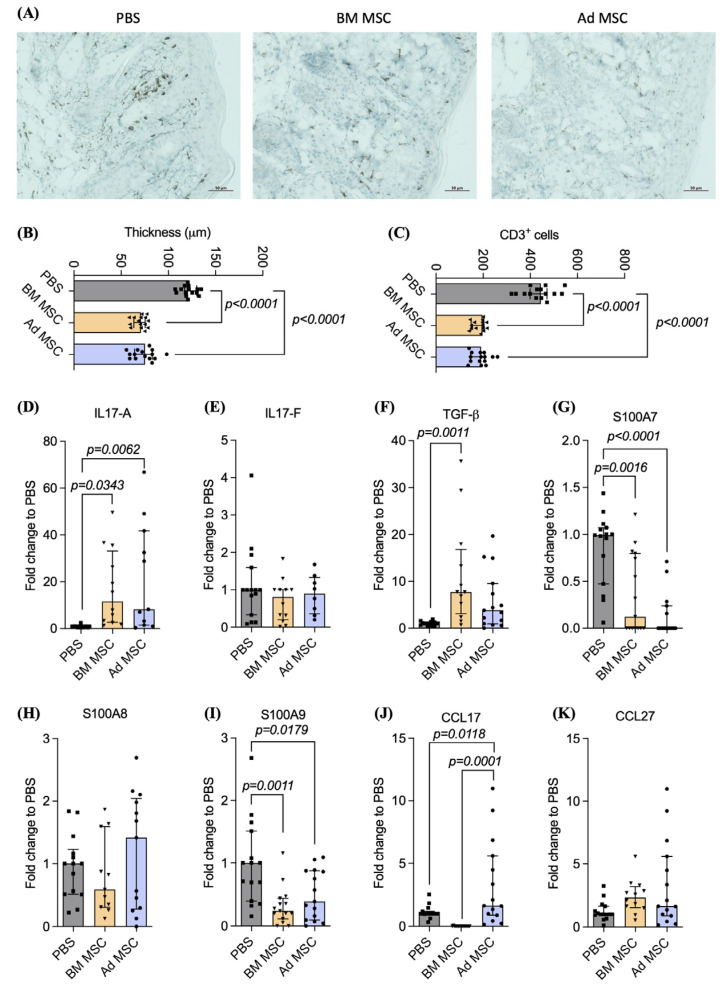
MSC infusion reduces epidermal thickness and CD3^+^ cell infiltration through the upregulation of cytokines and the downregulation of keratinocyte differentiation markers. (**A**) Typical presentation of histological analysis of infiltrating CD3^+^ T cells. Scale bar 50 μm. (**B**) Measurements of epidermal thickness (**C**) and CD3^+^ T cells of the mouse back skin. Relative gene expression of (**D**) *IL-17A*, (**E**) *IL-17F*, (**F**) *TGF-β*, (**G**) *S100A7*, (**H**) *S100A8*, (**I**) *S100A9*, (**J**) *CCL17* and (**K**) *CCL27* in the skin on Day 7. Five mice were used per experimental group and experiment was repeated three times. Mice infused with PBS, BM MSC or Ad MSC are represented in grey, yellow or blue, respectively. Data shown are the cumulative of the three independent experiments. Kruskal–Wallis test (also known as one-way ANOVA) coupled with Dunn’s multiple comparisons was performed to analyze statistical significance.

Histological analysis of the IMQ-treated skin for each group (Figure 2A) enabled the measurement of epidermal thickness (Figure 2B) and quantification of CD3^+^ T cell infiltration (Figure 2C) in the back of the skin. Briefly, infusion of BM or Ad MSC resulted in a significant reduction in both epidermal thickening (PBS: 120.7 [IQR 117.2–130.0]; BM MSC: 70.86 [IQR 63.34–75.35]; *p* < 0.0001; Ad MSC: 75.2 [IQR 64.2–82.8]; *p* < 0.0001) and T cell infiltration (PBS: 444.9 [IQR 399.1–471.3]; BM MSC: 196.8 [IQR 149.3–199.5]; *p* < 0.0001; Ad MSC: 191.2 [IQR 147.0–207.1]; *p* < 0.0001). The tissue source of MSC isolation had no significant effect on the thickness of the epidermis or the number of CD3^+^ T cells present. 

qRT-PCR of the IMQ-treated skin of mice was used to assess the effect of MSC administration on the expression of inflammatory mediators and keratinocyte differentiation factors closely related to psoriasis. Infusion of BM or Ad MSC resulted in a significant upregulation of *IL17-A* (PBS: 1 [IQR 0.4–1.2]; BM MSC: 12 [IQR 2.7–33]; *p* = 0.0343; Ad MSC: 8.2 [IQR 1.5–42]; *p* = 0.0062; Figure 2D) compared to infusion of PBS. MSC infusion had no significant effect on *IL17-F* levels (Figure 2E). The tissue source of MSC isolation had no significant effect on the expression of *IL17-A* and *IL17-F* levels in the skin. The infusion of BM MSC significantly upregulated *TGF-β* expression in the skin compared to PBS-treated control mice (PBS: 1.0 [IQR 0.6–1.2]; BM MSC: 7.7 [IQR 3.0–16.7]; *p* = 0.0011); Ad MSC infusion (Ad MSC: 3.8 [IQR 0.9–9.5]) did not result in a significant *TGF-β* upregulation compared to PBS control mice (Figure 2F). However, no significant differences were found in the expression of *TGF-β* in the skin of mice infused between BM and Ad MSC. 

The skin of the mice infused with BM or Ad MSC had significantly downregulated the expression of *S100A7* (PBS: 0.9 [IQR 0.4–1.4]; BM MSC: 0.1 [IQR 0.0–0.7]; *p* = 0.0016; Ad MSC: 0.0 [IQR 0.0–0.2]; *p* < 0.001; Figure 2G) and *S100A9* (PBS: 1.0 [IQR 0.3–1.5]; BM MSC: 0.2 [IQR 0.1–0.4]; *p* = 0.0011; Ad MSC: 0.3 [IQR 0.09–0.8]; *p = 0.0179;* Figure 2I) compared to control PBS mice. However, MSC infusion had no effect on *S100A8* (Figure 2H) expression. The tissue source of MSC isolation had no significant effect on the expression of *S100A7*, *S100A8* and *S100A9* in the skin.

The infusion of BM MSC resulted in a non-significant downregulation of expression of the Treg-attracting chemokine *CCL17* (Figure 2J) while the infusion of Ad MSC resulted in the upregulation of *CCL17* expression in the skin compared to PBS control mice (PBS: 1 [IQR 1.0–1.1]; Ad MSC: 1.6 [IQR 0.8–5.5]; *p* = 0.0118). Infusion of Ad MSC resulted in increased *CCL17* expression in the skin compared to BM MSC infusion (BM MSC: 0.61 ± 2.03; Ad MSC: 0.0 [IQR 0.0–0.06]; *p* = 0.0001). Infusion of BM or Ad MSC had no effect on *CCL27* expression in the skin (Figure 2K).

To sum up, infusion of BM or Ad MSC resulted in reduced skin thickness and CD3^+^ T cell infiltration compared to control mice that received a PBS infusion. In addition, the expression of *IL17-A* and *TGF-β* was upregulated in the skin on MSC-treated mice, while *S100A7* and *S100A9* were downregulated compared to the control.

### 2.3. MSC Licensing Decreases the Therapeutic Potential for the Treatment of Psoriasis-like Skin Inflammation

BM and Ad MSC were licensed with IFN-γ, TNF-α and IL-1β and flow cytometric analysis was used to study the effect of the licensing cytokines on the expression of CD73 (Figure S1A), CD146 (Figure S1B), CD166 (Figure S1C), CD271 (Figure S1D), MHC Class I (Figure S1E) and MHC Class II (Figure S1F). Under unlicensed conditions, CD73 and CD146 were expressed by 98.6 (IQR 97.9–98.7) and 92.5 (IQR 90.9–94.4) of BM MSC and 99.3 (IQR 98.7–99.5) and 92.9 (IQR 92.7–98.6) of Ad MSC; 22.9 (IQR 21.8–23.6) of BM MSC and 35.1 (IQR 33.5–38.1) of Ad MSC expressed CD166. CD271 and MHC Class I were expressed by 41.2 (IQR 36.4–43.0) and 24.5 (IQR 23.0–28.1) of BM MSC, respectively, while 3.2 (IQR 3.2–4.6) of Ad MSC expressed CD271 and 64.8 (IQR 61.5–67.2) expressed MHC Class I under unlicensed conditions (Figure S1G). Under unlicensed conditions, 1.7 (IQR 1.1–2.1) and 0.4 (IQR 0.2–1.2) of BM and Ad MSC, respectively, expressed MHC Class II. Licensing of BM (BM MSC +) and Ad MSC (Ad MSC +) resulted in no significant variation of CD73 and CD271 mesenchymal markers while significantly increasing MHC Class I expression (BM MSC +: 48.3 [IQR 46.2–51.0]; *p* < 0.0001; Ad MSC +: 77.8 [IQR 75.4–80.2]; *p* < 0.0001) and MHC Class II expression (BM MSC +: 5.1 [QR 4.8–5.5]; *p* < 0.0001; Ad MSC +: 1.5 [IQR 0.8–1.6]; *p* < 0.0001) (Figure S1G). Licensing did not affect CD146 and CD166 expression by BM MSC; however, licensed Ad MSC had significantly decreased expression of CD146 (Ad MSC +: 80.5 [IQR 79.6–83.4]; *p* = 0.0004) and CD166 (Ad MSC +: 27.6 [IQR 24.5–31.6]; *p* = 0.0004). 

**Figure 3:**
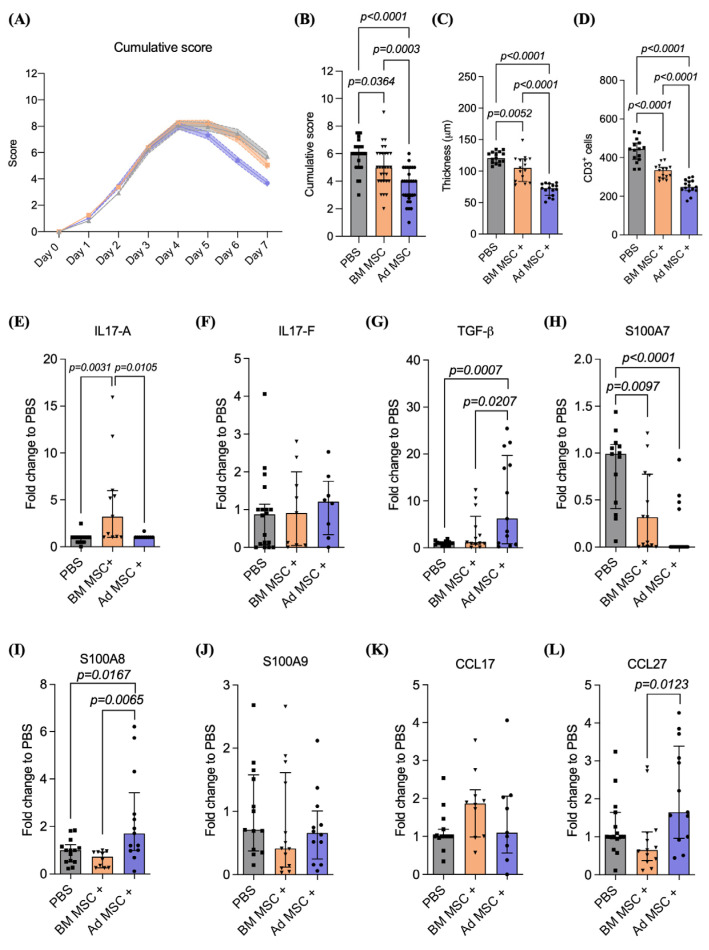
MSC licensing decreases the therapeutic potential of MSC for the treatment of imiquimod-induced psoriasis-like skin inflammation. (**A**) Cumulative score (0 to 12) and (**B**) cumulative score on day 7 of mice infused with PBS (grey), licensed BM (orange) or Ad MSC (violet). (**C**) Measurements of epidermal thickness (**D**) and CD3^+^ T cells of the mouse back skin. Relative gene expression of (**E**) *IL-17A*, (**F**) *IL-17F*, (**G**) *TGF-β,* (**H**) *S100A7*, (**I**) *S100A8*, (**J**) *S100A9*, (**K**) *CCL17* and (**L**) *CCL27* in the skin on Day 7. Five mice were used per experimental group and experiment was repeated three times. Data shown are the cumulative of the three independent experiments. Kruskal–Wallis test (also known as one-way ANOVA) coupled with Dunn’s multiple comparisons was performed to analyze statistical significance.

IMQ was topically applied to the back of the mice daily for four consecutive days prior to intravenous (IV) administration of licensed BM or Ad MSC (1 × 10^6^ cells/mouse in 100 μL of PBS) or PBS and mice were monitored daily for skin symptoms. On day 4, 24 h after the last IMQ application (+/− licensed MSC infusion), the PASI score stopped increasing and the skin started to heal in all groups, as represented by the gradual decrease in the cumulative PASI score (Figure 3A). PBS-injected mice healed slowly and still had a mean cumulative PASI score of 6.0 (IQR 5.0–6.5) on day 7 when the experiments were terminated. The infusion of licensed BM or Ad MSC significantly decreased the cumulative PASI score measured on day 7 compared to PBS-infused mice (BM MSC +: 5.0 [IQR 4.0–6.0]; *p* = 0.0364; Ad MSC +: 4.0 [IQR 2.8–5.0]; *p* < 0.0001; Figure 3B). However, the infusion of licensed Ad MSC resulted in a significantly lower cumulative PASI score compared to infusion of licensed BM MSC (*p* = 0.0003). The licensing of Ad MSC resulted in a significant decrease in erythema compared to PBS control (Figure S2A,B; PBS: 2.0 [IQR 1.5–2.0]; Ad MSC +: 1.0 [IQR 1.0–1.5]; *p* = 0.0078). No significant differences were observed between the erythema scores of the back of the mice infused with PBS or licensed BM, or Ad MSC. The infusion of licensed BM or Ad MSC resulted in a significant reduction of scaling (PBS: 2.0 [IQR 2.0–2.5]; BM MSC +: 1.5 [IQR 1.0–2.0]; *p* = 0.0097; Ad MSC +: 1.0 [IQR 1.0–1.8]; p < 0.0001; Figure S2C,D). No significant differences were observed in scaling scores between mice infused with licensed BM or Ad MSC. Infusion of licensed Ad MSC resulted in a significant thickening reduction compared to control PBS-infused mice (PBS: 2.0 [IQR 2.0–2.3]; Ad MSC +: 1.0 [IQR 1.0–2.0]; *p* = 0.0010) or licensed BM MSC-infused mice (BM MSC +: 2.0 [IQR 1.5–2.0]; *p* = 0.0123; Figure S2E,F). Infusion of licensed BM MSC had no effect on thickening. Typical skin recovery patterns observed on day 7 for licensed BM and Ad MSC are illustrated in Figure S2G. Representative histology of the skin of mice infused with licensed BM or Ad MSC can be found in Figure S2H. Day 0 to day 7 progression and cumulative score as well as erythema, scaling and thickening scores, epidermal thickness and number of CD3^+^ T cells among PBS, control and licensed BM or Ad MSC infused mice is depicted in Figure S3. A similarity matrix comparing the cumulative score of all treatment groups can be found in Table 1. 

Histological analysis of the IMQ-treated skin for each group (Figure S2H) enabled measurement of the epidermal thickness (Figure 3C) and quantification of CD3^+^ T cell infiltration (Figure 3D) in the skin of mice infused with licensed BM and Ad MSC. Infusion of licensed BM or Ad MSC resulted in a significant reduction in epidermal thickness compared to PBS (PBS: 120.4 [IQR 113.7–130.0]; BM MSC +: 105.1 [IQR 83.64–119.3]; *p* = 0.0052; Ad MSC +: 72.85 [IQR 61.28–79.59]; *p* < 0.0001). Infusion of licensed Ad MSC significantly decreased skin thickness compared to infusion of licensed BM MSC (*p* < 0.0001). Infusion of licensed BM or Ad MSC resulted in a significant reduction of T cell infiltration compared to mice infused with PBS (PBS: 444.5 [IQR 390.3–475.7]; BM MSC +: 334.1 [IQR 293.9–349.2]; *p* < 0.0001; Ad MSC +: 247.6 [IQR 228.7–279.1]; *p* < 0.0001). The tissue source of MSC isolation had a significant effect on the number of CD3^+^ T cells present in the skin, where the infusion of licensed Ad MSC resulted in a reduction in CD3^+^ T cells present in the skin compared to infusion of licensed BM MSC (*p* < 0.0001).

The infusion of licensed BM MSC resulted in a significant upregulation of *IL17-A* compared to the infusion of PBS (Figure 3E; PBS: 1.0 [IQR 0.4–1.0]; BM MSC +: 3.2 [IQR 1.0–5.9]; *p* = 0.0031) or licensed Ad MSC (Ad MSC +: 1.0 [IQR 1.0–1.0]; *p = 0.0105*). The infusion of licensed BM or Ad MSC had no effect on *IL17-F* levels (Figure 3F). The infusion of licensed Ad MSC resulted in a significant upregulation of *TGF-β* compared to the infusion of PBS (Figure 3E; PBS: 1.0 [IQR 0.6–1.2]; Ad MSC +: 6.2 [IQR 0.8–19.6]; *p* = 0.0007) or licensed BM MSC (BM MSC +: 1.1 [IQR 0.7–6.7]; *p* = 0.0207). The skin of the mice infused with licensed BM or Ad MSC had significantly downregulated expression of *S100A7* (Figure 3H; PBS: 0.9 [IQR 0.4–1.0]; BM MSC +: 0.3 [IQR 0.01–0.7]; *p* = 0.0097; Ad MSC +: 0.001 [0.0001–0.1]; *p* < 0.0001) compared to mice infused with control PBS. Infusion of licensed Ad MSC resulted in a significant upregulation of *S100A8* compared to infusion of PBS (Figure 3I; PBS: 1.0 [IQR 0.5–1.2]; Ad MSC +: 1.7 [IQR 0.9–3.4]; *p* = 0.0167) or licensed BM MSC (BM MSC +: 0.7 [IQR 0.2–0.9]; *p* = 0.0065). Licensed MSC infusion, regardless of tissue source of isolation, had no effect on *S100A9* (Figure 3J) or *CCL17* (Figure 3K) expression. The infusion of licensed BM or Ad MSC had no effect on *CCL27* (Figure 3L) expression in the skin compared to the infusion of PBS. However, infusion of licensed Ad MSC resulted in higher *CCL27* expression compared to infusion of licensed BM MSC (BM MSC +: 0.6 [IQR 0.3–1.1]; Ad MSC +: 1.6 [IQR 0.9–3.3]; *p* = 0.0123).

In conclusion, the infusion of licensed BM or Ad MSC resulted in decreased thickness and decreased CD3^+^ T cell infiltration compared to control; however, MSC licensing, regardless of the tissue’s source of isolation, resulted in the decreased therapeutic potential of MSC for the treatment of IMQ-induced psoriasis-like skin inflammation.

### 2.4. Angiogenic, Regenerative and Immunomodulatory Potential of MSC Influence the Therapeutic Outcome

The mechanisms underlying the in vivo differences observed in the pro-resolution potential of the MSC preparations were investigated by assaying the transcription and protein secretion profile of immunomodulatory, pro-angiogenic and chemoattractant molecules of BM and Ad MSC under unlicensed and licensed conditions. 

**Figure 4:**
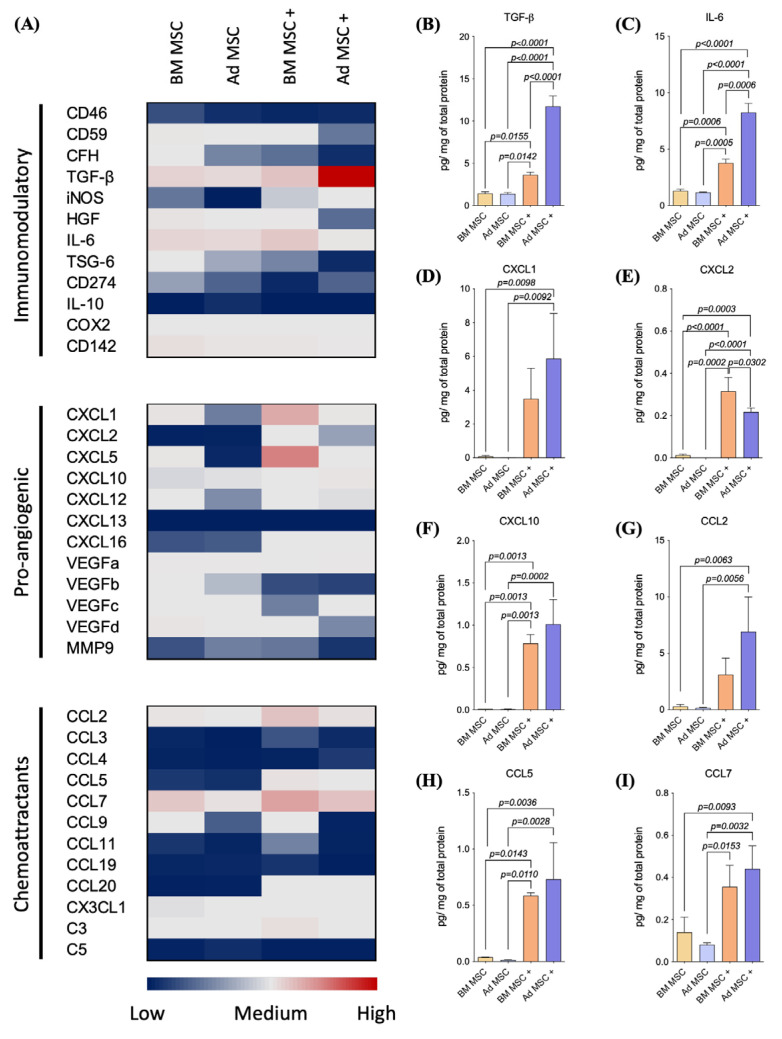
Angiogenic, immunomodulatory and chemoattractant potential of MSC at transcriptional and protein levels. (**A**) Gene expression of unlicensed and licensed BM and Ad MSC (BM MSC = yellow; BM MSC + = orange; Ad MSC = blue; Ad MSC + = violet). The expression of immunomodulatory, pro-angiogenic, and chemoattractant genes was measured by qRT-PCR. (**B**) Secreted protein levels of TGF-β, (**C**) IL-6, (**D**) CXCL1, I CXCL2, (**F**) CXCL10, (**G**) CCL2, (**H**) CCL5 and (**I**) CCL7 were detected by Luminex in 24 h supernatants harvested from unlicensed and licensed (+) BM and Ad MSC cultures. Data shown are the cumulative of three independent experiments. Data represents picogram of protein of interest per mg of protein. Kruskal–Wallis test (also known as one-way ANOVA) coupled with Dunn’s multiple comparisons was performed to analyze statistical significance.

Unlicensed BM and Ad MSC expressed similar transcript patterns of genes involved in the initiation and control of inflammation (Figure 4A), which correlated with the similar outcome observed in the IMQ-mediated psoriasis-like inflammation model. However, MSC licensing (+) resulted in a small upregulation of *TGF-β, iNOS* and *IL-6* in BM MSC, and a pronounced upregulation in Ad MSC. MSC tissue source of isolation and licensing had little to no effect in *CD46*, *CD59*, *CD142*, *CFH*, *HGF*, *TSG-6*, *CD274* and *IL-10* transcription in BM and Ad MSC. 

At the protein level, unlicensed BM and Ad MSC secreted moderate amounts of TGF-β (BM MSC: 1.4 [IQR 1.2–1.6] pg/mg of protein; Ad MSC: 1.3 [IQR 1.2–1.5] pg/mg of protein), while licensing significantly increased TGF-β secretion in MSC from both sources (BM MSC +: 3.5 [IQR 3.2–3.9] pg/mg of protein; *p* = 0.0155; Ad MSC +: 11.9 [IQR 10.3–12.8] pg/mg of protein; *p* < 0.0001) (Figure 4B). While unlicensed BM and Ad MSC secreted similar amounts of TGF-β, licensed Ad MSC secreted higher amounts of TGF-β than licensed BM MSC (*p* < 0.0001). Similarly, unlicensed BM and Ad MSC secreted moderate amounts of IL-6 (BM MSC: 1.3 [IQR 1.0–1.4] pg/mg of protein; Ad MSC: 1.1 [IQR 1.0–1.2] pg/mg of protein), while licensing significantly increased IL-6 secretion in MSC from both sources (BM MSC +: 3.7 [IQR 3.4–4.0] pg/mg of protein; *p* = 0.0006; Ad MSC +: 8.3 [IQR 7.3–8.9] pg/mg of protein; *p* < 0.0001) (Figure 4C). While unlicensed BM and Ad MSC secreted similar amounts of IL-6, licensed Ad MSC secreted higher amounts of IL-6 than licensed BM MSC (*p* = 0.0006).

Unlicensed BM and Ad MSC expressed similar transcript patterns of the pro-angiogenic factors *CXCL2*, *CXCL10*, *CXCL13*, *CXCL16*, *VEGFa*, *VEGFb*, *VEGFc*, *VEGFd* and *MMP9* (Figure 4A). Unlicensed BM MSC expressed higher levels of *CXCL1*, *CXCL5* and *CXCL12* than Ad MSC. Licensing upregulated *CXCL1*, *CXCL2*, *CXCL5*, *CXCL10* and *CXCL16* and downregulated *VEGFb* in MSC from both sources. Single licensing upregulated *CXCL12* in Ad MSC only and downregulated *VEGFc* in BM MSC only. MSC licensing had little to no effect in *CXCL13*, *VEGFa*, *VEGFd* and *MMP9* in MSC from both sources and it had no effect in *VEGFc* in Ad MSC. At the protein level, unlicensed BM and Ad MSC secreted similar levels of CXCL1 (BM MSC: 0.1 [IQR 0.01–0.1] pg/mg of protein; Ad MSC: 0.01 [IQR 0.01–1–0.013] pg/mg of protein; Figure 4D), CXCL2 (BM MSC: 0.01 [IQR 0.007–0.01] pg/mg of protein; Ad MSC: 0.0001 [IQR 0.000009–0.0001] pg/mg of protein; Figure 4E) and CXCL10 (BM MSC: 0.01 [IQR 0.01–0.01] pg/mg of protein; Ad MSC: 0.009 [IQR 0.007–0.01] pg/mg of protein; Figure 4F). Licensing upregulated the secretion of CXCL1 by Ad MSC (Ad MSC +: 6.2 [IQR 3.0–8.3] pg/mg of protein; *p* = 0.0092). Licensing upregulated the secretion of CXCL2 (BM MSC +: 0.3 [IQR 0.2–0.4] pg/mg of protein; *p* < 0.0001; Ad MSC +: 0.2 [IQR 0.1–0.2] pg/mg of protein; *p* < 0.0001) and CXCL10 (BM MSC +: 0.8 [IQR 0.6–0.8] pg/mg of protein; *p* = 0.0013; Ad MSC +: 1.0 [IQR 0.6–1.2] pg/mg of protein; *p* = 0.0022) in MSC from both sources. Licensed Ad MSC secreted significantly less CXCL2 than licensed BM MSC (*p* = 0.032). No significant differences were found in the secretion of CXCL1 and CXCL10 between licensed BM and Ad MSC.

Unlicensed BM and Ad MSC expressed similar transcript patterns of CC chemokines and *CX3CL1* and the chemoattractant factors *CKLF*, *C3* and *C5* (Figure 4A). Licensing upregulated the transcription of *CCL2*, *CCL5*, *CCL7* and *CCL20* in MSC from both tissue sources; Ad MSC transcribed very high levels of *CCL7* upon licensing. At the protein level, unlicensed BM and Ad MSC secreted similar levels of CCL2 (BM MSC: 0.3 [IQR 0.09–0.4] pg/mg of protein; Ad MSC: 0.1 [IQR 0.1–0.2] pg/mg of protein; Figure 4G), CCL5 (BM MSC: 0.03 [IQR 0.03–0.04] pg/mg of protein; Ad MSC: 0.01 [IQR 0.008–0.01] pg/mg of protein; Figure 4H) and CCL7 (BM MSC: 0.1 [IQR 0.05–0.1] pg/mg of protein; Ad MSC: 0.08 [IQR 0.07–0.09] pg/mg of protein; Figure 4I). MSC licensing resulted in the increased secretion of CCL2 (BM MSC +: 3.7 [IQR 1.4–1.0] pg/mg of protein; *p* = 0.0063; Ad MSC +: 8.3 [IQR 3.3–8.9] pg/mg of protein; *p* = 0.0056) and CCL5 (BM MSC +: 0.5 [IQR 0.5–0.6] pg/mg of protein; *p* = 0.0143; Ad MSC +: 0.8 [IQR 0.3–0.9] pg/mg of protein; *p* = 0.0028) regardless of tissue source of isolation. Licensing only increased CCL7 secretion in Ad MSC (Ad MSC +: 0.4 [IQR 0.3–0.5] pg/mg of protein; *p* = 0.0032). No significant differences were found in the secretion of CCL2, CCL5 and CCL7 between licensed BM and Ad MSC. 

Tissue source of isolation had little to no effect on the transcriptional profiling of immunomodulatory, pro-angiogenic or chemoattractant genes; however, MSC licensing drastically upregulated the expression and secretion of TGF-β and IL-6. 

## 3. Discussion

In this study, we have evaluated the therapeutic potential of Ad MSC for the treatment of IMQ-induced psoriasis-like skin inflammation and compared it to the gold standard BM MSC. Furthermore, we have evaluated the cytokine profiling at transcriptome and protein levels to elucidate the mechanism behind BM and Ad MSC therapeutic potential. In addition, we have described that licensing with a cocktail of IFN-γ, TNF-α and IL-1β does not improve the therapeutic potential of MSC in this disease model, in fact, it decreased it; the therapeutic potential of MSC may be predicted by the analysis of the angiogenic, chemoattractant and immunomodulatory cytokine profiling of the cells. 

Our results showed that the infusion of Ad MSC accelerated the healing response and sharply reduced the severity of psoriasis, measured as erythema, scaling, thickening and CD3^+^ T cell infiltration to the same levels as the infusion of gold standard BM MSC. Psoriatic skin is characterized by downregulation of TGF-β; TGF-β normally inhibits the growth of keratinocytes and reduced TGF-β levels in the skin potentiate keratinocyte hyperproliferation [42]. Herein, we describe that improved recovery of the skin was associated with increased *IL-17A* and *TGF-β* in the skin of mice treated with BM or Ad MSC, and we hypothesize that TGF-β promoted the controlled differentiation of keratinocytes, resulting in the decreased severity of psoriasis. This statement is supported by the significant reduction in keratinocyte differentiation promoters *SA100A7* and *SA100A9*. Furthermore, we propose that TGF-β is also responsible for reduced CD3^+^ T cell infiltration upon MSC treatment. TGF-β has a pivotal role in controlling T cell homeostasis; TGF-β inhibits Th1 and Th2 cell differentiation whereas TGF-β deficiency in mice results in a lethal T cell-dependent multifocal inflammatory disease because of spontaneous T cell activation [43,44]. 

The upregulation of TGF-β could be seen as having mixed effects in psoriasis as TGF-β also promotes T cell differentiation towards Th17 phenotypes [45], where T cell and neutrophil-derived IL-17A is seen as a pivotal pro-psoriatic cytokine [46] and therapeutic agents targeting IL-17 or anti-IL-17 monoclonal antibodies have been proven to be effective in the treatment of psoriasis [47,48]. However, in our control groups, despite measurable disease and skin T cell infiltrate, *IL-17A* was significantly decreased compared to MSC-treated groups, while *IL-17F* was similar to MSC-treated groups. We hypothesize that in this model, the removal of the IMQ insult quickly removes the IL-17A expression in the skin, but we unequivocally show that IL-17A is associated with more rapid healing in the presence of MSC. IL-17A is a potent activator of the immunological function of MSC [49,50], hence, it is possible that this represents a mechanism for MSC to co-opt IL-17A in order to prolong or enhance their anti-inflammatory function. Furthermore, the Th17 response is regulated by an autocrine loop where binding of IL-17A to its own receptor represses the Th17 response [51], which correlates with the increased IL-17A levels and decreased CD3^+^ T cell infiltration described in the skin of the mice infused with BM or Ad MSC. 

There are a variety of case reports which show potential for MSC in the treatment of psoriasis, but the number of patients treated is very small (eight across these four studies, and one using conditioned medium rather than cells [22,23,52,53]). Nevertheless, this would point to an anti-inflammatory secretome from the MSC having an anti-psoriatic effect [50]. This study contradicts this to an extent since accelerated healing is associated with increased expression of both IL-17A and TGF-β in the skin. In our study, we found that both BM and Ad MSC express and secrete high levels of the broadly pro-inflammatory cytokine IL-6. IL-6 has been associated with the pathogenesis of psoriasis and increased levels of this cytokine in the skin and serum is a characteristic of this disease [54,55]. Moreover, IL-6 levels are positively correlated with clinical severity and effective treatment of psoriasis results in a reduction of IL-6 levels [56]. However, IL-6 has a dual role in the modulation of inflammation and here we describe that IL-6 secreting MSC are correlated with the resolution of inflammation. IL-6 promotes anti-inflammatory Th2 cell differentiation [57] and increases IL-27 secretion by monocytes and macrophages promoting the maturation of regulatory T cells [58]. Furthermore, in the presence of IL-6, TGF-β induces the development of Th17 [45]. Recently, two distinct populations of Th17 cells with differential secretion profiles have been described. TGF-β promotes IL-10 expression in Th17 cells, which acquire an anti-inflammatory phenotype able to impair the pro-inflammatory reaction of effector Th17 cells [59]. Thus, IL-6 secretion by MSC may form part of the TGF-β and IL-17A axis associated with healing in this model. 

Several attempts have been made to increase the potency of MSC therapy. In 2006, Krampera et al. showed the importance of IFN-ɣ licensing to boost MSC immunoregulatory properties. TNF-α and IL-1β, in combination with IFN-ɣ, have been described to produce a synergistic effect to further enhance the immunomodulatory properties of MSC by decreasing pro-inflammatory markers and producing an increase in anti-inflammatory markers [31,33]. 

Here, we describe that licensing of autologous MSC decreased the therapeutic potential of MSC; interestingly, licensing differentially affected the therapeutic potential of BM and Ad MSC to dampen inflammation in the IMQ model. Infusion of licensed BM MSC resulted in a significantly diminished potential with similar scores to control untreated animals while licensed Ad MSC were as effective in decreasing epidermal thickening and CD3^+^ T cells as unlicensed Ad or BM MSC but did not achieve the same levels of healing in terms of erythema, scaling and the overall cumulative score. 

An increasing number of studies have shown that immunomodulatory and pro-angiogenic phenotypes correlated with positive outcomes and that cytokine profiling enables the prediction of leukocyte recruitment, immunomodulatory potential and T cell inhibition potential and thus, the therapeutic outcome [49,51]. Tobin et al. demonstrated that the difference in efficiency between unlicensed and licensed MSC for the treatment of stroke was based on the differential MSC secretome. In their scenario, the hallmarks of oligodendrocyte precursor cells and the hallmarks of maturation of myelinating oligodendrocytes were only present in the licensed MSC, which resulted in an improved pro-oligodendrogenic response upon treatment with licensed MSC compared to unlicensed MSC [60]. Thus, to investigate the differences between the therapeutic potential of BM and Ad MSC under unlicensed and licensed conditions, we studied the transcription and secretion of immunomodulatory and pro-angiogenic factors as well as chemoattractants. 

Resting unlicensed BM and Ad MSC showed very similar immunomodulatory, pro-angiogenic and chemoattractant profiles, with MSC from both sources secreting moderate levels of IL-6 and high levels of TGF-β. However, MSC licensing results in increased IL-6 and TGF-β secretion by Ad MSC only and increased neutrophil chemoattractant CXCL1, CXCL2 and CXCL10 and monocyte/macrophage chemoattractants CCL2, CCL5 and CCL7 by MSC from both sources. We postulate that the recruitment of neutrophils and monocytes/macrophages hampered the healing of the skin, explaining the increased erythema and scaling observed in the skin of the mice infused with licensed MSC compared to unlicensed MSC infusion. Decreased CXCL2 secretion by licensed Ad MSC compared to licensed BM MSC correlated with decreased erythema and scaling in mice infused with licensed Ad MSC compared to licensed BM MSC, which further suggests that increased neutrophil recruitment upon MSC licensing might have hampered the healing of the skin. Another key difference between licensed BM and Ad MSC is the upregulation of IL-6 and TGF-β secretion, which have the potential to restrain T cell-mediated pathology and could be the cause of the decrease in thickness and the number of CD3^+^ T cells (similar to those of mice infused with unlicensed BM or Ad MSC) in mice infused with licensed Ad MSC compared to infusion of licensed BM MSC. 

To exert their therapeutic potential upon infusion into an inflammatory environment, MSC must resist senescence as well as maintain their immunomodulatory properties, which depend on the balance of signals in the new microenvironment. Ad MSC have increased resistance to oxidative stress and hypoxia compared to BM MSC as a result of the increased electron transport chain activity and reactive oxygen species (ROS) production in the adipose tissue [30,61]. We hypothesize that accumulation of ROS generates an inflammatory environment that influences the microenvironment of Ad MSC, which as opposed to BM MSC, enables Ad MSC to better maintain their pro-resolution properties upon licensing and infusion into the IMQ-induced psoriasis-like skin inflammation model [62]. 

In our particular scenario, the IMQ-induced psoriasis-like inflammation model is characterized by the downregulation of TGF-β and thus, the high transcription and secretion of TGF-β can potentially alleviate the disorder. However, for the treatment of fibrosis, where there is a pathologic excess of tissue fibrosis that compromises the normal function of the tissue or organ, high levels of TGF-β would be detrimental as it promotes the accumulation of extracellular matrix proteins, and thus, promotes fibrosis [63]. Thus, pro-resolution phenotypes for specific disorders could be predicted using cytokine and chemokine profiling. 

The results of this study must be considered within its own limitations. First and probably most importantly, the etiology and pathogenesis of psoriasis is multifactorial and involves genetic, immunological, and environmental factors. While the IMQ model effectively induces psoriasis-like skin inflammation, it does not capture the complex immunopathology and genetic background observed in human psoriasis. Furthermore, the strain of mice used could also affect the impact of psoriasiform inflammation. Thus, findings from this study should be interpreted with caution when extrapolating to human disease and future work should consider evaluation of MSC infusion using intradermal injection of IL-23. The PASI score has been extensively used to evaluate the treatment efficacy of psoriasis; however, the distinction between active psoriatic lesions and residual discoloration or scarring can be challenging, which can potentially introduce variability and affect the accuracy of PASI assessment. In addition, it involves subjective assessments of erythema, scaling, and thickness which can affect the consistency and reliability of PASI scores, compromising its objectivity and comparability across studies. To avoid variability in PASI scoring within this study, a single individual, blinded, scored all the animals used in this study. Basal culture media composition, the addition of serum or serum-free supplements as well as the oxygen culture environment impact the immunomodulatory potential of MSC, which difficult inter-study comparisons. Future studies should focus on the development of standardized culture protocols to maximize the immunomodulatory potential of MSC. Finally, mechanistic studies, including the blockage of the IL-6/TGF-β axis, would be required to depict the mechanism by which MSC alleviates the imiquimod-mediated psoriasis-like skin inflammation. 

In conclusion, we have described that intravenous administration of autologous Ad MSC has therapeutic potential for the treatment of skin inflammation and thus, could have potential applications for the treatment of psoriasis. Furthermore, we have proposed that the mechanism could be based on TGF-β and IL-6 secretion, which have the potential to restrain T cell-mediated pathology through the upregulation of TGF-β and IL-17A in the skin. Lastly, we have described that MSC licensing alters the secretome of MSC and decreases the therapeutic potential of MSC for the alleviation of IMQ-induced psoriasis-like skin inflammation. However, different disorders will require distinct immunomodulatory and pro-regenerative factors to achieve resolution, and therefore, cytokine profiling could enable determining which tissue source of MSC isolation is more therapeutically beneficial for a particular scenario as well as to predict the effect of licensing on the pro-resolution phenotype of MSC.

## 4. Materials and Methods

### 4.1. Culture Medium

MSC were isolated and grown in culture medium containing Dulbecco’s Modified Eagle Medium (Sigma, Glasgow, UK) with high glucose and sodium pyruvate (Invitrogen) supplemented with 20% (*v*/*v*) heat-inactivated fetal calf serum (FCS) (Invitrogen, Paisley, UK) and 2 mM glutamine (Sigma) (“MSC Culture medium”). Cells were maintained and grown in a humidified incubator at 37 °C and 5% CO_2_.

### 4.2. Isolation of Bone Marrow MSC

C57BL/6 female mice aged 7 weeks were sacrificed with cervical dislocation and sterilized with 70% ethanol. The skin from the hind limbs was removed; muscles, ligaments and tendons were dissociated, and tibias and femurs were dissected from the trunk of the body. The knee joint and the ends of the tibia and femurs were cut through. A 27 G needle attached to a 10 mL syringe containing complete medium was inserted into the spongy bone and was used to flush the bone marrow out, which was collected in 100 mm sterile Petri dishes (Fisher).

Petri dishes containing the bone marrow were incubated at 37 °C with 5% CO_2_ for 5 days, after which MSC were recovered with a 2-min incubation at 37 °C with 0.13 mL/cm^2^ Trypsin (Thermo Fisher Scientific, Paisley, UK). Cells were counted and designated as “passage 1” (P1). Cells were then resuspended into 0.26 mL/cm^2^ of culture medium and distributed at a density of 3500 cells/cm^2^ in Corning CellBIND flasks. Cultures were examined daily for growth using a Zeiss optical microscope and media was changed twice per week. Upon reaching 80–90% confluency, cells were recovered with a 10-min incubation at 37 °C with 0.13 mL/cm^2^ TrypLE™ Express Enzyme (Thermo) for detachment. Cell debris was removed by centrifugation at 400× *g* for 5 min, cells were counted using a hemocytometer and were either preserved at 1 × 10^6^ cells/mL in Cellbanker cell freezing media (Amsbio) or resuspended into 0.26 mL/cm^2^ of culture medium and distributed at a density of 3500 cells/cm^2^ in Corning CellBIND flasks. 

### 4.3. Isolation of Adipose MSC

Animals were sacrificed as above. A midline incision was made around the abdomen and the skin was retracted using straight tweezers. The muscular wall was then opened to expose the liver and intestines. Perigonadal adipose tissue was harvested. Adipose tissue was digested using 0.2 mg/mL Collagenase P (Roche, London, UK) and 0.1 mg/mL DNAse (Roche) in HBSS for 40 min at 37 °C. After incubation, collagenase was inactivated using medium and the soft tissues were plated into 100 mm sterile Petri dishes (Fisher). Petri dishes were incubated as above and MSC were passaged as above. 

### 4.4. MSC Licensing

When cultures achieved a cell density >80% confluence, the medium was discarded, and cells were washed twice with DPBS. For single licensing, MSC culture medium was replaced with licensing MSC medium (MSC culture medium supplemented with 40 ng/mL IFN-γ, TNF-α and interleukin (IL)-1β [Peprotech]) and cells were harvested 24 h later. For double licensing, the MSC culture medium was replaced with licensing MSC medium, 48 h later cells were washed with DPBS and fresh licensing MSC medium was provided; cells were harvested 24 h later. For in vivo experiments, unlicensed and single-licensed MSC were used.

### 4.5. Flow Cytometry

MSC were harvested as above and then dissociated into a single cell suspension and washed twice in buffer comprising 2% FCS, and 2 mM EDTA in DPBS (flow buffer) prior to staining using antibodies at various concentrations (detailed in Table S1) in a total volume of 100 μL for 20 min at 4 °C. Cells were washed in flow buffer and resuspended in 200 μL of flow buffer for analysis. A minimum of 10,000 events were collected. Flow cytometry acquisition was performed using BD LSRFortessa (BD Biosciences) or MACS Quant and analyzed with Flow Jo V10 software for Windows (BD BioSciences, Ashland, OR, USA).

### 4.6. Imiquimod-Induced Psoriasis-like Skin Inflammation Model

Seven-week-old C57BL/6 female mice with an average weight of 19.0 ± 1.0 g received 62.5 mg Aldara cream (Aldara™, MEDA Ab) containing 5% IMQ on their shaved backs for four consecutive days. Five animals were used per experimental group and experiments were repeated three times in all cases. The sample size was calculated as follows:

(1) $$\mathrm{Sample size}=\frac{2\cdot {SD}^{2}\cdot ({Z_{\alpha /2}+Z_{\beta })}^{2}}{d^{2}}=1.32\approx 2\;\mathrm{mice per group}$$

Standard deviation (SD) = 1.103 was calculated from a pilot study

Z_α/2_ = Z _0.05/2_ = Z _0.025_ = 1.96 (From Z table) at type 1 error of 5% 

Z_β_ = Z _0.20_ = 0.842 (From Z table) at 80% power 

d = effect size = difference between mean values = 3.848

(2) $$\mathrm{Corrected sample size}=\frac{\mathrm{Samples size}}{1-(\%\;\mathrm{of attraction})}=2.22\approx 3\;\mathrm{mice per group}$$

% of attrition = 10% to compensate for the potential death of the mice during the experiment. 

Five animals were used per group instead of three due to the 10–20% reported failure rate of intraperitoneal injections. 

The severity of the psoriasis-like skin inflammation was assessed using the Psoriasis Area and Severity Index (PASI) to objectively score the severity of inflammation of the back skin. The PASI score combines assessments of erythema, scaling, and thickening, and assigns a numerical value from 0 to 4 (0, none; 1, slight; 2, moderate; 3, severe and 4, very severe) to each parameter based on its severity. Erythema assessment entails the comparison of psoriatic lesions to adjacent normal skin to evaluate the intensity and extent of the color of psoriatic lesions. Scaling assessment entails a macroscopic evaluation of the skin to record the presence, degree and proportion of scaling or flaky skin in psoriatic lesions. Thickening was measured using a semi-quantitative scoring system to visually grade the degree of thickening in comparison to control skin. The cumulative score was calculated through the sum of erythema, scaling, and thickening scores.

### 4.7. MSC Adoptive Therapy

The effects of MSC were tested by administration of 1 × 10^6^ unlicensed or licensed BM or Ad MSC in 0.1 mL of sterile PBS or sterile PBS alone (control mice) via the tail vein on the last day of IMQ treatment; 1 × 10^6^ cell dose was chosen based on previous studies 16; 96 h later, mice were euthanized using a recognized Schedule 1 technique (CO_2_ followed by femoral artery exsanguination) and skin samples were collected. 

### 4.8. Skin Histology and Immunohistochemical Analysis

Skin samples were fixed in 10% neutral buffered formalin prior to processing and paraffin embedding. Processing was performed using the Shandon Citadel 1000 automated tissue processor (Thermo Fisher Scientific). Immunohistochemical analysis for CD3 was performed on 5-μm-thick paraffin-embedded sections from sequential regions through the skin. Briefly, upon rehydration, antigen retrieval was performed using Dako Target retrieval solution (pH 6; Agilent Technologies, Cheadle, UK) for 45 min at 95 °C. Dako peroxidase blocking reagent was used to block endogenous peroxidase for 10 min, and non-specific binding was blocked for 20 min with Dako protein block. The primary antibody rabbit anti-mouse CD3 antibody (Vector, Peterborough, UK) was added and incubated for 1 h at room temperature. An isotype-matched irrelevant antibody was used as a negative control. Slides were then washed three times with Tris-buffered saline and were incubated for 30 min with peroxidase-conjugated Dako EnVision polymer. After three further washes, diaminobenzidine chromogen (Agilent Technologies) was used to visualize peroxidase activity and slides were lightly counterstained with hematoxylin before mounting in DePex (VWR International, Leicestershire, UK). The number of immune-reactive cells was determined by counting positively stained cells on photomicrographs obtained from three random microscopic fields (20× magnification) under a Zeiss epi-fluorescent microscope. Images were processed using Zenn microscopy software V2.1 (Zeiss, Jena, Germany).

Counterstained hematoxylin enabled measurement of the thickness of the epidermis using the Zeiss software; three measurements were taken per section and three sections were analyzed per mouse. Hematoxylin staining was used to discriminate the epidermis based on nuclear size and shape, nuclear density and basal layer. The nuclei of the epidermal cells exhibited a more uniform and smaller size compared to the nuclei of dermal cells. Epidermal nuclei tend to be relatively small, round to oval in shape, and tightly packed, enabling discrimination from dermal cells. The nuclei within the epidermis are more densely packed compared to the nuclei in the dermis due to the close arrangement of epidermal cells, forming a distinct layer with minimal intercellular space. The basal layer of the epidermis, which is the bottommost layer in contact with the basement membrane, displays a unique nuclear pattern where the nuclei are larger and more elongated compared to the cells in the suprabasal layers. 

### 4.9. Gene Expression

Total RNA extraction from mouse back skin was performed with the miRNeasy Mini Kit (Qiagen, Manchester, UK) and RNA was reverse transcribed by QuantiTect Reverse Transcription Kit (Qiagen) according to the manufacturer’s instructions. MSC were licensed as previously described, or left as unlicensed controls, and then were harvested as above. Supernatants were frozen at −80 °C for Luminex analysis of protein expression (see below). Shortly, RNA extraction was performed with the RNeasy Mini Kit (Qiagen) and RNA was reverse transcribed by QuantiTect Reverse Transcription Kit (Qiagen) according to the manufacturer’s instructions. In both cases, pairs of primers were designed (detailed in Table S2) to relatively quantify the amount of specific cDNA in a sample by SYBR Green (QuantaBio). The quantitative reverse transcription PCR assay was performed using the Applied Biosystems 7900HC Fast Real-Time PCR Systems detection system (Applied Biosystems, Paisley, UK). 

Samples were analyzed using *TBP* as a reference for data normalization and data were represented as 2^(−ΔΔCT)^. 

### 4.10. Protein Secretion

Conditioned media from the same samples used for transcript analysis were collected and analyzed using a Luminex 100 analyzer (Bio-Rad, Hertfordshire, UK) and premixed magnetic multianalyte kits (R&D systems, Abingdon, UK) in accordance with the manufacturer’s instructions. All reagents and standards were included in the kit and prepared as outlined in the guidelines. Briefly, samples were diluted two-fold with calibrator diluent (75 μL in 75 μL); 10 μL of the pre-coated microparticle cocktail was added to each well of the 96 well microplates, followed by either a 50 μL sample or 50 μL standard, sealed and placed on an orbital shaker (0.12 mm orbit at 800 ± 50 rpm) for 2 h at room temperature (RT). The plates were washed twice with 100 μL/well wash buffer and then incubated with 50 μL/well anti-biotin detector antibody for 1 h at RT on the shaker (0.12 mm orbit at 800 ± 50 rpm). The plates were washed as before and 50 μL/well of streptavidin-phycoerythrin was added and incubated for 30 min at RT. Microparticles were resuspended in 100 μL/well of wash buffer and immediately read on the Bio-Rad analyzer. Each microparticle bead region was designated and doublets were excluded as stated on the certificate of analysis.

### 4.11. Statistics

Graphs and statistical analysis were generated with GraphPad Prism 8 software for Windows (GraphPad, Boston, MA, USA). Results are shown as mean ± standard deviation (SD) unless stated otherwise. Unpaired two-tailed Student’s *t*-tests were used to compare BM vs. Ad MSC and when assessing statistical differences within one tissue source. If more than two groups were compared, a Kruskal–Wallis test coupled with Dunn’s multiple comparisons test was used to evaluate statistical significance. Pearson’s correlation coefficients were used to generate the correlation matrix.

## Figures and Tables

**Table 1:** Similarity matrix comparing the cumulative score of all treatment groups.

	PBS	BM MSC	Ad MSC	BM MSC +	Ad MSC +
PBS	1.00	0.18	0.14	0.58	0.33
BM MSC	0.18	1.00	0.45	0.08	−0.11
Ad MSC	0.14	0.45	1.00	−0.06	0.17
BM MSC +	0.58	0.08	−0.06	1.00	0.39
Ad MSC +	0.33	−0.11	0.17	0.39	1.00

## Data Availability

The datasets generated and/or analyzed during the current study are available from the corresponding author upon reasonable request. This paper does not report original code and did not generate new unique reagents.

## Supplementary Materials

The following supporting information can be downloaded at: https://www.mdpi.com/article/10.3390/ijms241210132/s1.
