# Supplementary material for: IL-6 and TGF-β-Secreting Adoptively-Transferred Murine Mesenchymal Stromal Cells Accelerate Healing of Psoriasis-like Skin Inflammation and Upregulate IL-17A and TGF-β
Source: Int J Mol Sci. 2023 Jun 14;24(12):10132. doi: 10.3390/ijms241210132 (PMC10298958; doi:10.3390/ijms241210132)
Supplement: Supplementary file 1 [file ijms-24-10132-s001.zip › ijms-2442521-supplementary.pdf]

IL-6 and TGF-β-secreting adoptively-transferred murine mesenchymal stromal cells accelerate healing of psoriasis-like skin inflammation and upregulate IL-17A and TGF-β

Nerea Cuesta-Gomez [1], Laura Medina-Ruiz [1], Gerard J Graham [1,!], John D M Campbell [1,2,*,!]

Supplementary figures

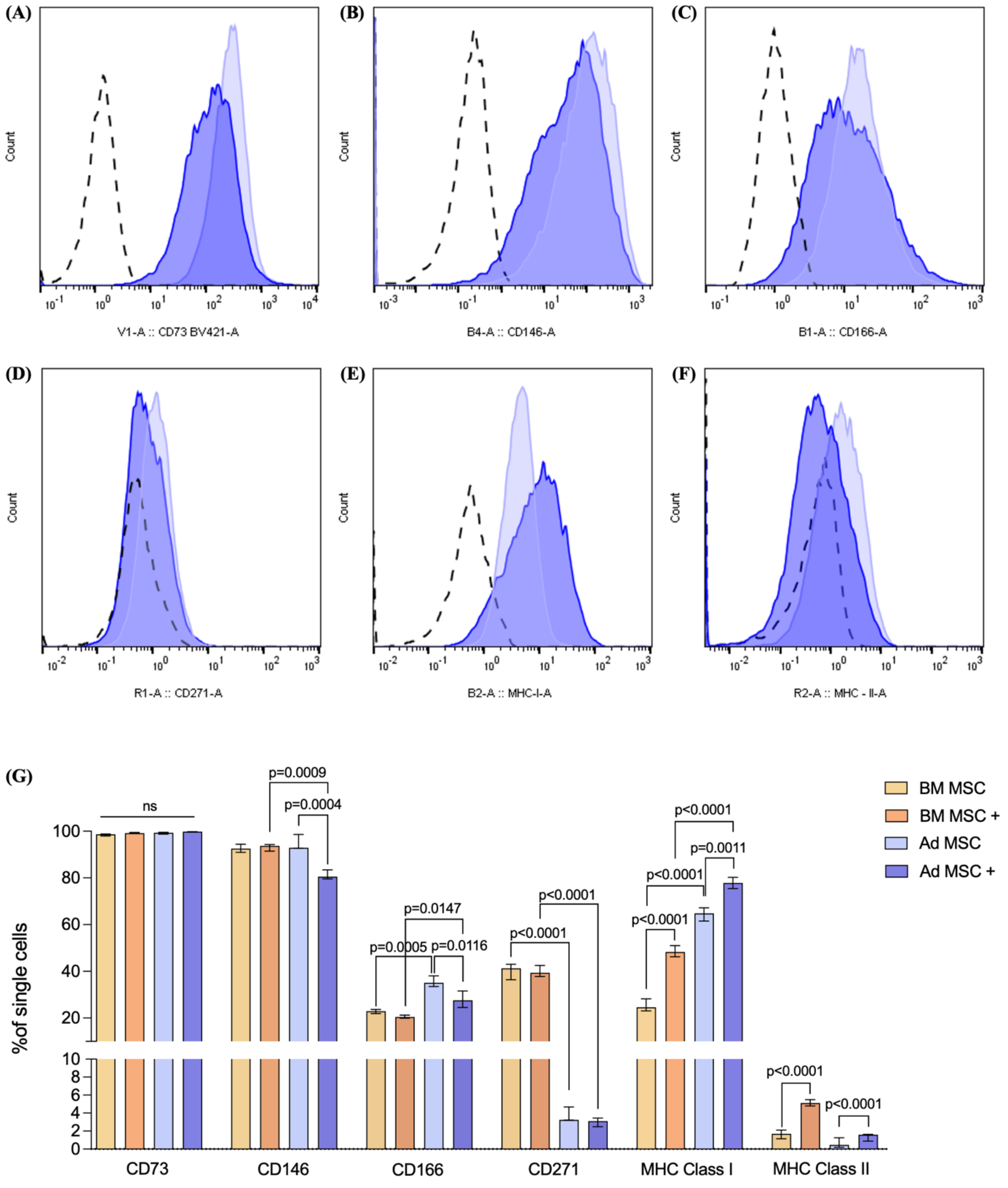

Figure S1. MSC characterization after licensing with a cocktail of IFN-γ, TNF-α and IL-1β. (**a**) Representative gating strategy for the quantification of CD73, (**b**) CD146, (**c**) CD166, (**d**) CD271, (**e**) MHC Class

I and (f) MHC Class II. A dashed line was used to represent the isotype while blue and violet were used to represent un-licensed and licensed Ad MSC, respectively. (**g**) Quantification of the percentage of single cells that stained positive for the above-mentioned markers.

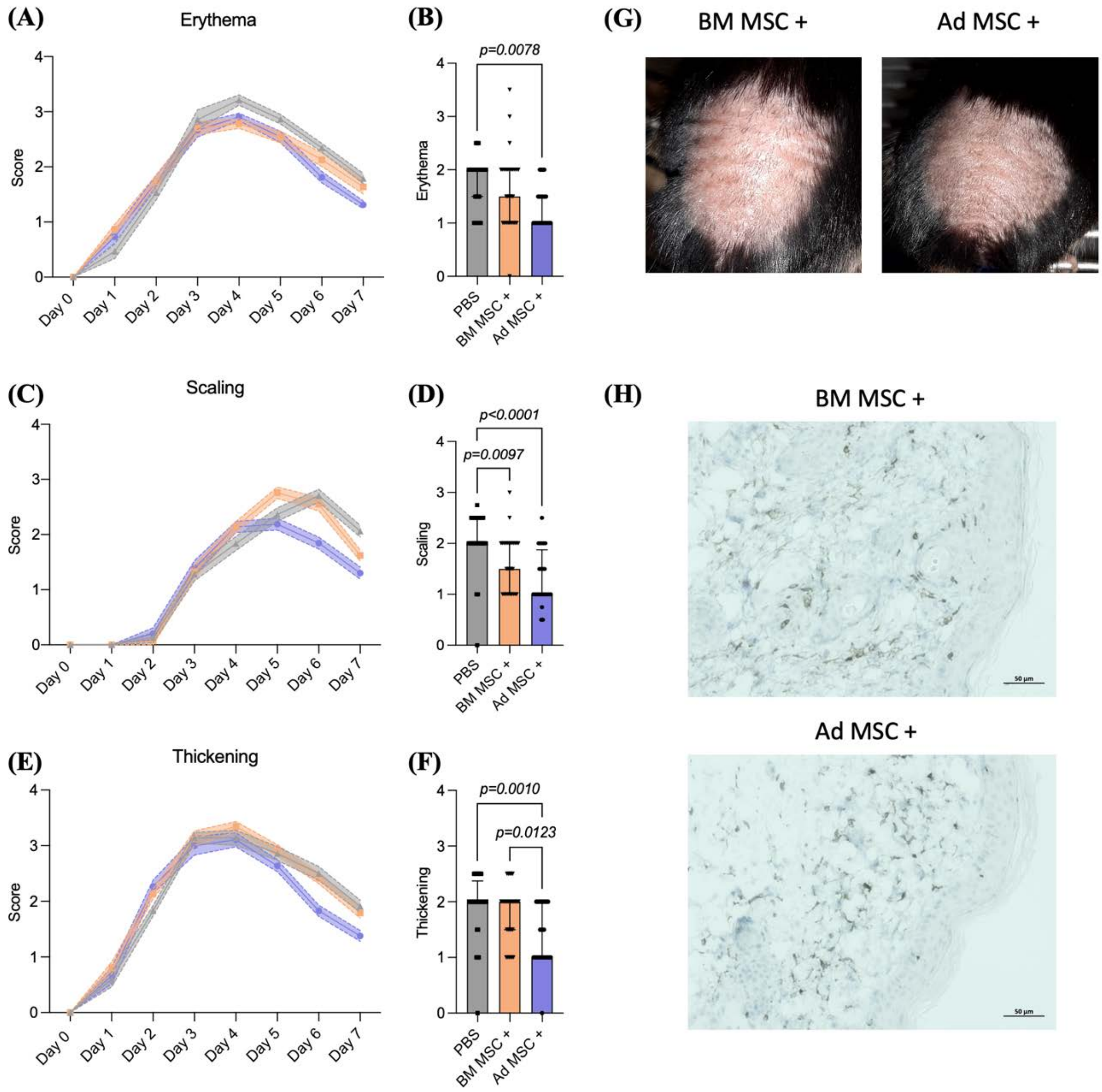

Figure S2. Licensed MSC infusion reduced epidermal thickening and $CD3^+$ T cell infiltration. (a) Erythema, (c) scaling and (e) thickening of the skin of mice infused with PBS (grey) or licensed BM (orange) or Ad MSC (violet) were scored on a scale from 0 to 4 as follows: 0, none; 1, slight; 2, moderate; 3, severe; and 4, very severe. PASI scored on day 7 graphed for (b) erythema, (d) scaling and (f) thickening. (g) Typical presentation of the skin 7 days after first IMQ application. (h) Typical presentation of IHC analysis of infiltrating $CD3^+$ T cells. Scale bar 50μm. 5 mice were used per experimental group and experiment was repeated three times. Data shown is the cumulative of the three independent experiments. Kruskal-Wallis test (also known as one-way ANOVA) coupled with Dunn's multiple comparison was performed to analyze statistical significance.

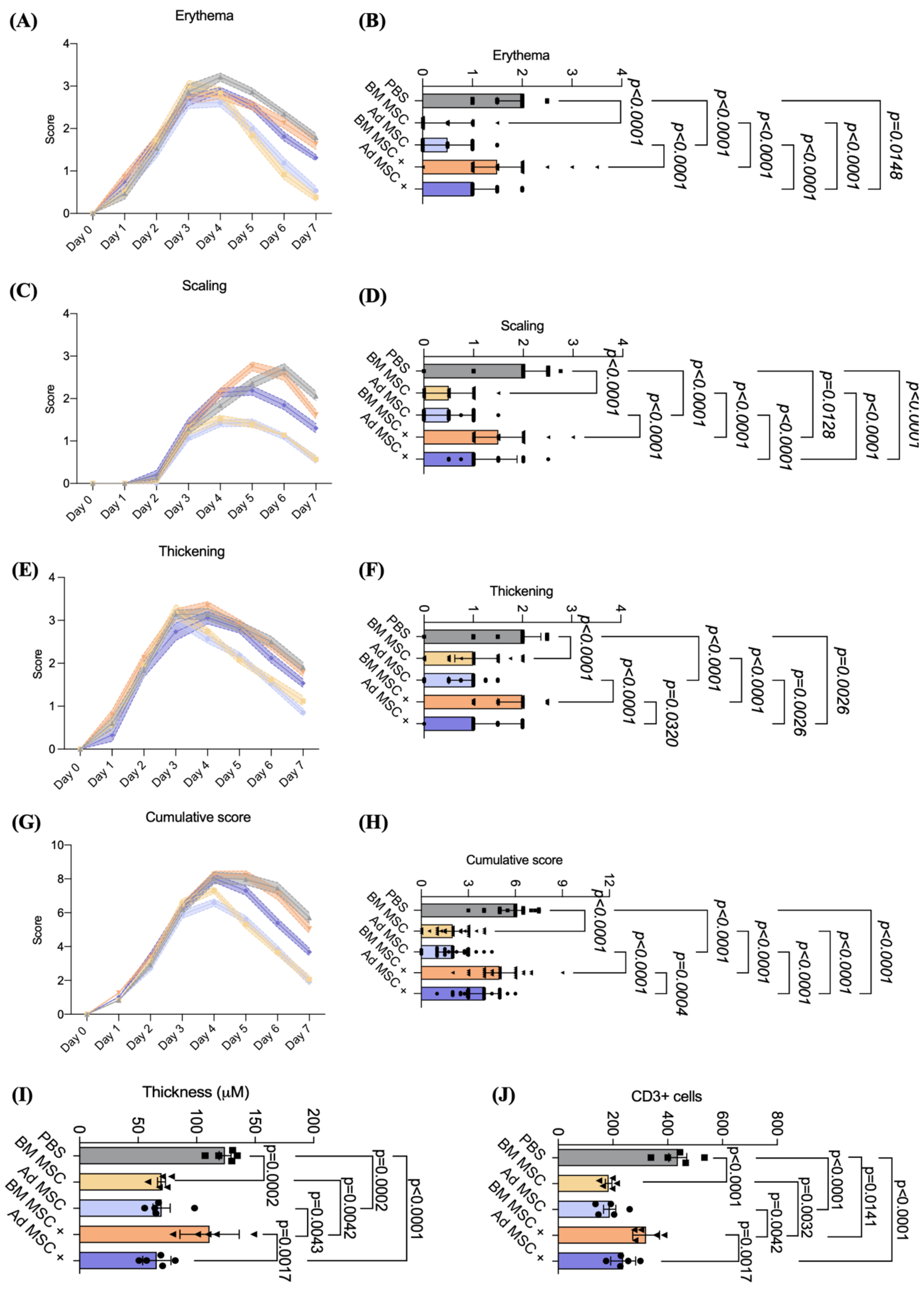

Figure S3. MSC licensing reduces the therapeutic potential for the treatment of imiquimod-mediated psoriasis-like skin inflammation. (**a**) Erythema, (**c**) scaling, (**e**) thickening and (**g**) cumulative score of the skin of mice infused with PBS (grey) or resting or licensed BM or Ad MSC were scored on a scale from 0 to 4 as

follows: 0, none; 1, slight; 2, moderate; 3, severe; and 4, very severe (BM MSC = yellow; BM MSC + = orange; Ad MSC = blue; Ad MSC + = violet). PASI scored on day 7 graphed for (**b**) erythema, (**d**) scaling (**f**) thickening and (**h**) cumulative score. (**i**) Measurements of epidermal thickness (**j**) and $CD3^+$ T cells of the mouse back skin.

Supplementary methods

Table S1. Monoclonal Antibodies for flow cytometry.

**Antigen**	**Fluorophore**	**Clone**	**Dilution**	**Supplier**
CD73	BV 421	TY/11.8	1:200	BioLegend
Fixable viability dye	eFluor 506		1:1000	eBioscience
CD166	FITC	eBioALC48	1:200	eBioscience
MHC Class I	PE	M1/42	1:200	BioLegend
CD146	PE /Cy7	ME-9F1	1:200	BioLegend
CD 271	APC	REA648	1:200	Miltenyi
MHC Class II	APC – Cy7	M5/114.15.2	1:200	BioLegend

Table S2. Primer sequences for gene expression analysis

**Gene**	**Forward primer**	**Reverse primer**
IL17A	TCTCTGATGCTGTTGCTGCT	ACGTGGAACGGTTGAGGTAG
IL17F	GTGTTCCCAATGCCTCACTT	CTCCTCCCATGCATTCTGAT
TGF- β	CTTTGTACAACAGCACCCGC	CATAGATGGCGTTGTTGCGG
CCL17	AGTGGAGTGTTCCAGGGATG	GGTCACAGGCCGTTTTATGT
CCL27	TTTCCTTGGCTGCGAATGTGGC	CTTGTTCCACGGATGCCAGCTT
CXCL2	CCTCAACGGAAGAACCAAAG	AGGCACATCAGGTACGATCC
S100A7	GCCTCGCTTCATGGACAC	CGGAACAGCTCTGTGATGTAGT
S100A8	TCCTTGCGATGGTGATAAA	GGCCAGAAGCTCTGCTACTC
S100A9	GACACCCTGACACCCTGAG	TGAGGGCTTCATTTCTCTTCTC
B2M	GGTGACCCTGGTCTTTCTGG	TGTTCGGCTTCCCATTCTCC
CCL2	AGCCAACTCTCACTGAAGCC	GCGTTAACTGCATCTGGCTG
CCL3	CAGCCAGGTGTCATTTTCCT	CAGGCATTCAGTTCCAGGTC
CCL4	TGACCAAAAGAGGCAGACAGA T	GCTGTGCCACATCTCTTGGT
CCL5	CTGCTGCTTTGCCTACCTCT	ACACACTTGGCGGTTCCTT
CCL7	TGAAAACCCCAACTCCAAAG	TTAGGCGTGACCATTTCACA
CCL9	CTCACAACCACGGACCTACA	CACTGGGGAAGACCAAAGAA
CCL11	GCACCCTGAAAGCCATAGTCT	TGGGGTCAGCACAGATCTCT
CCL19	GTGCCTGCTGTTGTGTTCAC	CAAGACACAGGGCTCCTTCTG

Gene	Forward primer	Reverse primer
CCL20	CGACTGTTGCCTCTCGTACA	CTTCATCGGCCATCTGTCTT
CXCL1	CCGAAGTCATAGCCACACTCA	AGGTGCCATCAGAGCAGTCT
CXCL5	GCCCTACGGTGGAAGTCATA	GTGCATTCCGCTTAGCTTTC
CXCL10	GCTCAAGTGGCTGGGATG	GAGGACAAGGAGGGTGTGG
CXCL12	CCTCAACCCACCATGCTCAT	GAGACAGTCTTGCGGACACA
CXCL13	CATACCCAACCCACATCCTT	GCCTGTTCTCAAATAGCCTTTC
CXCL16	TGCTGACCCTTTGCCTCTAC	GGCTGGCTTGGACTAAATAACA
CX3CL1	CAACTTCCGAGGCACAGGAT	AGATGTCAGCCGCCTCAAAA
C3	TGCTGGCCTCTGGAGTAGAT	AGGCAGTCTTCTTCGGTGTG
C5	CCTGCTGAAGCCCAAGAGAA	GCAGGGTGTTTTCAAGCAGG
CD46	GGAGCTCTTATCCCCATGCC	GACTGAGTGTGGAAGGCACA
CD59	TGGTAGCCCAGCACAATGAG	TGTGAGGCTAACAGCTGTGG
CFH	ACAACGGGTTTTCACCACCT	GTGCAACGAAGGTAGTCCCA
CFI	GCGGGGGTAGTGTGTTACAA	TCGCTTTGGTCTCCACAGTC
CR1L	GGATTCCAGAAGGGGTTGGG	TTCCAGCTGCCATCAGACTG
CD274	CAGCAACTTCAGGGGGAGAG	CTGTGATCTGAAGGGCAGCA
HGF	TGAGTTATGTGCTGGGGCTG	CACATCCACGACCAGGAACA
IDO 1	TGGTGGAAATCGCAGCTTCT	TTGACGCTCTACTGCACTGG
IDO 2	ACCTCCCTCGTCCCTTAGTC	AGAGAGTAAGCAGGGGAGGG
IFNβ	CACAGCCCTCTCCATCAACT	GCATCTTCTCCGTCATCTCC
IL-10	CAGAGAAGCATGGCCCAGAA	GCTCCACTGCCTTGCTCTTA
IL-6	TTCCATCCAGTTGCCTTCTT	ATTTCCACGATTTCCCAGAG
iNOS	GAGCCACAGTCCTCTTTGCT	CAACCTTGGTGTTGAAGG G
MMP9	AAACCCTGTGTGTTCCCGTT	CCTTTAGTGGTGCAGGCAGA
TSG6	CGGATACCCCATTGTGAAAC	TCCTTTGCATGTGGGTTGTA
VEGF a	AACGATGAAGCCCTGGAGTG	GCTGGCTTTGGTGAGGTTTG
VEGF b	AGAGTGCTGTGAAGCCAGAC	GATGATGTCAGCTGGGGAGG
VEGF c	AACCTCCATGTGTGTCCGTC	TGCTGAGGTAACCTGTGCTG
VEGF d	TTCAGGAGCGAACATGGACC	CCACAGCTTCCAGTCCTCAG
